# DIBR-Synthesized Image Quality Assessment With Texture and Depth Information

**DOI:** 10.3389/fnins.2021.761610

**Published:** 2021-11-03

**Authors:** Guangcheng Wang, Quan Shi, Yeqin Shao, Lijuan Tang

**Affiliations:** ^1^School of Transportation and Civil Engineering, Nantong University, Nantong, China; ^2^School of Electronics and Information, Jiangsu Vocational College of Business, Nantong, China

**Keywords:** depth-image-based-rendering, image quality assessment, colorfulness, texture structure, depth structure

## Abstract

Accurately predicting the quality of depth-image-based-rendering (DIBR) synthesized images is of great significance in promoting DIBR techniques. Recently, many DIBR-synthesized image quality assessment (IQA) algorithms have been proposed to quantify the distortion that existed in texture images. However, these methods ignore the damage of DIBR algorithms on the depth structure of DIBR-synthesized images and thus fail to accurately evaluate the visual quality of DIBR-synthesized images. To this end, this paper presents a DIBR-synthesized image quality assessment metric with Texture and Depth Information, dubbed as TDI. TDI predicts the quality of DIBR-synthesized images by jointly measuring the synthesized image's colorfulness, texture structure, and depth structure. The design principle of our TDI includes two points: (1) DIBR technologies bring color deviation to DIBR-synthesized images, and so measuring colorfulness can effectively predict the quality of DIBR-synthesized images. (2) In the hole-filling process, DIBR technologies introduce the local geometric distortion, which destroys the texture structure of DIBR-synthesized images and affects the relationship between the foreground and background of DIBR-synthesized images. Thus, we can accurately evaluate DIBR-synthesized image quality through a joint representation of texture and depth structures. Experiments show that our TDI outperforms the competing state-of-the-art algorithms in predicting the visual quality of DIBR-synthesized images.

## 1. Introduction

With the advent of the 5G era and the advancement of 3-dimensional display technology, video technology moves from “seeing clearly” to the ultra-high definition and immersive virtual reality era of “seeing the reality.” Free-viewpoint videos (FVVs) have broad applications in entertainment, education, medical treatment, military applications for its ability to provide users with visual information of integrity, immersion, and interactivity (Selzer et al., 2019; Yildirim, 2019). Thus, FVV is also regarded as the vital research direction of next-generation video technologies (Tanimoto et al., 2011). Due to hardware conditions, cost, and bandwidth constraints, it is feasible to collect a certain number of viewpoint images in realistic environments. Still, it is often impractical to collect a full range of 360-degree viewpoint images. Therefore, it is necessary to synthesize virtual viewpoint images from existing reference viewpoint images by relying on virtual viewpoint synthesis techniques (Wang et al., 2020, 2021; Li et al., 2021a; Ling et al., 2021).

**Figure 1:**
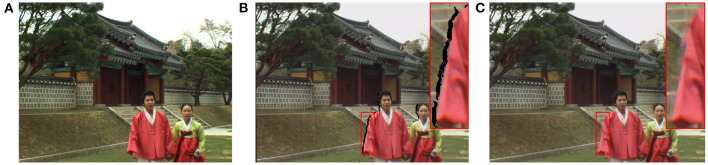
Examples of the local geometric distortion and the color deviation distortion in the synthesized images. **(A)** is the ground-truth image. **(B,C)** are the synthesized images, which includes the local geometric distortion and the color deviation distortion compared to the ground-truth image.

Because depth-image-based-rendering (DIBR) technologies only require a texture image and its corresponding depth map to generate the image at any viewpoint, it becomes the most popular virtual viewpoint synthesis technique (Luo et al., 2020). Unfortunately, because the performance of existing DIBR algorithms is not perfect, some distortions are often introduced during the warping and rendering processes, as shown in Figure 1. The quality of DIBR-synthesized images directly influences the visual experience in FVV-related applications, determining whether these applications can be successfully put into use. Hence, studying the quality evaluation methods for virtual viewpoint synthesis has important practical significance.

Image quality assessment (IQA) has been a crucial frontier research direction in image processing in recent decades. Massive IQA algorithms for natural images have been proposed, divided into full-reference, reduced-reference, and no-reference according to the required full, partial, and no information of the reference image. For instance, Wang et al. (2004) proposed a full-reference IQA metric based on comparing the structural information between the reference and distorted images, namely Structural SIMilarity (SSIM). Zhai et al. (2012) quantify psychovisual quality of images based on free-energy interpretation of cognition in brain theory. Min et al. (2018) proposed a pseudo-reference image (PRI) based IQA framework, which is different from the traditional full-reference IQA framework. The standard full-reference IQA framework assumes that the reference image is a high visual quality image. In contrast, the framework proposed by Min et al. assumes that the reference image suffers the most severe distortion in related applications. Based on the PRI-based IQA framework, Min et al. measures the similarity between the distorted image's and the PRI's structures to estimate blockiness, sharpness, and noisiness.

In recent years, researchers have realized that IQA algorithms for natural images have difficulty in estimating the geometric distortion prevalent in DIBR-synthesized images. For this problem, Bosc et al. (2011) calculated the difference map between the synthesized image and the reference image based on SSIM and adopted a threshold strategy to detect the disoccluded area in the synthesized image. Then, the quality score of a synthesized image is obtained by measuring the average structural similarity of the disoccluded region. Conze et al. (2012) used SSIM to generate a similarity map between the reference image and the synthesized image and further extracted the texture, gradient direction, and image contrast weighting maps based on the obtained similarity map to predict the synthesized image quality score. Stankovic et al. designed the Morphological Wavelet Peak signal-to-noise ratio (MW-PSNR) for assessing the synthesized image quality (Dragana et al., 2015b). Meanwhile, the authors proposed a simplified version of MW-PSNR called MW-PSNR-reduce (Dragana et al., 2015b), which only uses the PSNR value of the higher-level scale image to predict the synthesized image quality. For better performance, Stankovic et al. adopted morphological pyramid decomposition to replace the morphological wavelet decomposition in the above-mentioned MW-PSNR (Dragana et al., 2015b) and MW-PSNR-reduce (Dragana et al., 2015b), which successively produce MP-PSNR (Dragana et al., 2015a) and MP-PSNR-reduce (Dragana et al., 2016). Although these methods for the synthesized images have better performance than the IQA algorithms devised for natural images, their performance still misses the actual requirements.

Over the past few years, researchers have been aware of a close relationship between quantifying the local geometric distortion and the quality assessment of DIBR-synthesized images and the screen content images (Gu et al., 2017b). Gu et al. (2018a), Li et al. (2018b), Jakhetiya et al. (2019), and Yue et al. (2019) have arranged the idea in the design of DIBR-synthesized IQA methods, respectively. In literature (Gu et al., 2018a), Gu et al. adopted an autoregression (AR)-based local description operator to estimate the local geometric distortion. Specifically, the authors measure the local geometric distortion by calculating the reconstruction error between the synthesized image and its AR-based prediction. In literature (Jakhetiya et al., 2019), assumed that the geometric distortion behavior is similar to the outliers and further proved this hypothesis using ROR statistics based on the three-Sigma rule. Based on this view, the authors highlight the local geometric distortion through a median filter and further fuse these prominent distortions to assess the synthesized image quality.

Moreover, based on the local geometric distortion measurement, Yue et al. (2019)'s and Li et al. (2018b)'s methods introduce global sharpness estimation to predict the synthesized image quality. Yue et al. (2019) considered three major DIBR-related distortions, including the disoccluded region, the stretching region, and global sharpness. The authors first detect disoccluded regions by analyzing the local similarity. Then, the stretching regions are determined by combining the local similarity analysis and a threshold solution. Finally, the authors measure inter-scale self-similarity to estimate global sharpness. Li et al. (2018b) designed a SIFT-flow warping based disoccluded region detection algorithm. Then, the geometric distortion is measured by combining with the size and distortion intensity of local disoccluded areas. Moreover, a reblurring-based solution is developed to capture blur distortion. We find two critical problems from the above-mentioned DIBR-synthesized IQA methods. First, these methods ignore the influence of color deviation distortion on the visual quality of DIBR-synthesized images. Second, These methods only focus on estimating the geometric distortion and blur distortion from textured images without considering the local geometric distortion's adverse effects on the synthesized image's depth structure.

Inspired by these findings, we present a newly synthesized image quality assessment metric that combines Texture and Depth Information, namely TDI. Specifically, we adopt the colorfulness module proposed by Hasler and Suesstrunk (2003) to extract the color features of a synthesized image and its reference image (i.e., the ground-truth image) and then calculate the feature error to estimate the color deviation distortion. We perform discrete wavelet transform on the texture information of the synthesized image and its reference image and further calculate the similarity of the high-frequency subbands of a pair of synthesized and reference images. The similarity result is used to estimate the local geometric distortion and global sharpness. Meanwhile, we use SSIM to compute the structural similarity between the depth maps of a pair of synthesized and reference images to represent the effects of the local geometric distortion and blur distortion on the depth of field of the synthesized image. In addition, TDI develops a linear weighting scheme to fuse the obtained features. We verify the performance of our TDI metric on the public IRCCyN/IVC DIBR-synthesized image database Bosc et al. (2011), and the experimental results prove that our TDI metric performs better than the competing state-of-the-art (SOTA) IQA algorithms. Compared with the existing works, the highlights of the proposed algorithm mainly include two aspects: (1) we integrate the color deviation distortion caused by DIBR algorithms into the development of DIBR-synthesized view quality perception model; (2) This paper estimates the quality degradation brought by the local geometric distortion and blur distortion from the texture and depth information of the synthesized view.

The remaining chapters of this paper are organized as follows. Section 2 introduces the proposed TDI in detail. Section 3 compares our TDI with SOTA IQA metrics for natural and DIBR-synthesized images. Section IV summarizes the whole research.

## 2. Proposed Method

**Figure 2:**
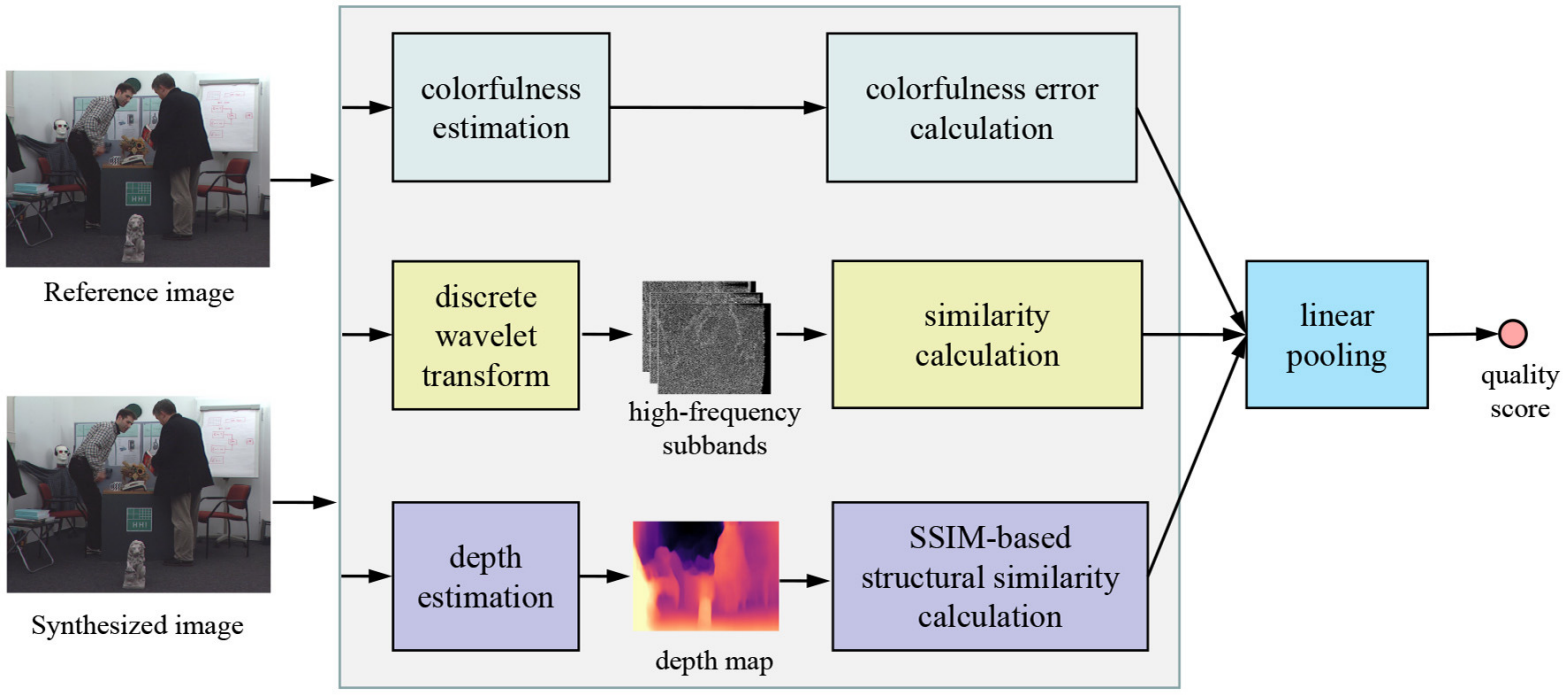
Framework of the proposed TDI metric for predicting the quality of DIBR-synthesized images.

The design philosophy of our TDI is based on quantifying the local geometric distortion, global sharpness, and color deviation distortion. After extracting the corresponding features, a linear weighting strategy fuses the above features to infer the final quality score. Figure 2 shows the framework of the proposed TDI.

### 2.1. Color Deviation Distortion Estimation

The human visual system (HVS) is susceptible to color, so the measurement of color deviation distortion has a direct impact on the visual experience (Gu et al., 2017a; Liao et al., 2019). As shown in Figure 1, compared to the high-quality reference image, the synthesized image has the color deviation distortion. However, since it is not the main distortion in the synthesized image, most existing DIBR-synthesized IQA algorithms ignore the impact of the color deviation distortion on the visual experience. To more accurately evaluate the synthesized image quality, this paper takes the measurement of color deviation distortion into account in the proposed TDI metric. In the literature (Hasler and Suesstrunk, 2003), Hasler and Suesstrunk devised a highly HVS-related image colorfulness estimation based on psychophysical category scale experiments. The image colorfulness estimation model is specifically defined as follows:


(1) $$\begin{array}{l} C={\left(\sigma_{rg}^{2}+\sigma_{yb}^{2}\right)}^{\frac{1}{2}}+0.3\cdot {\left(\mu_{rg}^{2}+\mu_{yb}^{2}\right)}^{\frac{1}{2}}, \end{array}$$

where σ_*rg*_, σ_*yb*_, μ_*rg*_ and μ_*yb*_ are the variance and mean of the *rg* and *yb* channels, respectively. The calculation method of *rg* and *yb* channels is shown in formula 2.


(2) $$\begin{array}{l} rg=R-G,yb=\frac{1}{2}\left(R+G\right)-B \end{array}$$

Then, we calculate the absolute value of the colorfulness difference between a synthesized image and its associated reference image (i.e., formula 5) as the quantized result of the color deviation distortion that existed in the synthesized image.


(3) $$\begin{array}{l} Q_{1}=|C_{syn}-C_{ref}|, \end{array}$$

where *C*_*syn*_ and *C*_*ref*_ represent the colorfulness of the synthesized image and its reference image, respectively.

### 2.2. Local Geometric Distortion and Global Sharpness Measurement

The proposed TDI extracts structural features from the texture image and its corresponding depth image and designs a linear pooling strategy for information fusion to achieve a more accurate measurement of the local geometric distortion and global sharpness. This part explains in detail how TDI extracts structure features from texture and depth images.

#### 2.2.1. Structure Feature Extracting From Texture Domain

**Figure 3:**
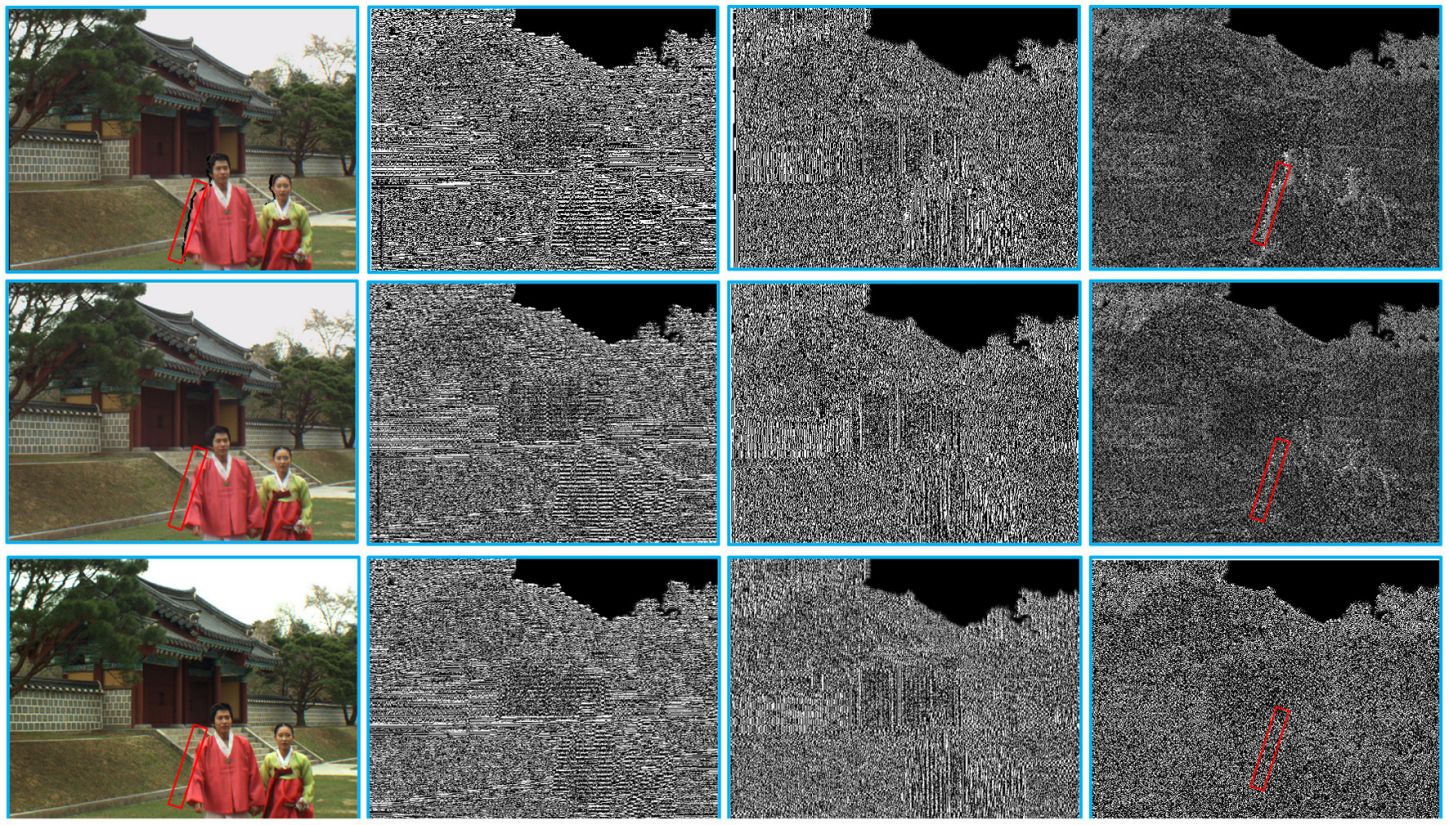
Examples of the high-frequency wavelet subbands (i.e., HL, LH, and HH subbands) of two synthesized images and their reference image. From left to right, the images in each row are a synthesized/reference image and its corresponding HL, LH, and HH wavelet subbands. Note that the synthesized image of the first row has only the warping process.

We first use the Cohen-Daubechies-Fauraue 9/7 filter (Cohen et al., 1992) to perform discrete wavelet transform on the synthesized and reference images. Figure 3 shows some examples of high-frequency wavelet subbands (i.e., HL, LH, and HH subbands) of two synthesized images and their reference image. From Figure 3, we observe that the geometric distortion regions (such as the red box area) of the synthesized and reference images in the HH subbands differ significantly. Motivated by this, we measure the local geometric distortion by computing the similarity between the HH subbands of a pair of synthesized and reference images, which is defined as follows:


(4) $$\begin{array}{l} Q_{2}=\frac{1}{N}\overset{N}{\underset{i=1}{\sum }}\left[\frac{2\cdot HH_{syn}\left(i\right)\cdot HH_{ref}\left(i\right)+\epsilon }{HH_{syn}\left(i\right)+HH_{ref}\left(i\right)+\epsilon }\right], \end{array}$$

where *HH*_*syn*_ and *HH*_*ref*_ represent the HH subbands of a synthesized image and its corresponding reference image. *i* and *N* are the pixel index and the number of pixels of a given image, respectively. A small constant ϵ avoids the risk of zero denominator. Moreover, since blur distortion usually causes loss of high-frequency information in images, the energy of high-frequency wavelet subbands has been widely used for no-reference image sharpness estimation (Vu and Chandler, 2012; Wang et al., 2020). Therefore, the developed similarity between the HH subbands of the synthesized image and its reference image can also effectively estimate the global sharpness of the DIBR-synthesized image.

#### 2.2.2. Structure Feature Extracting From Depth Domian

**Figure 4:**
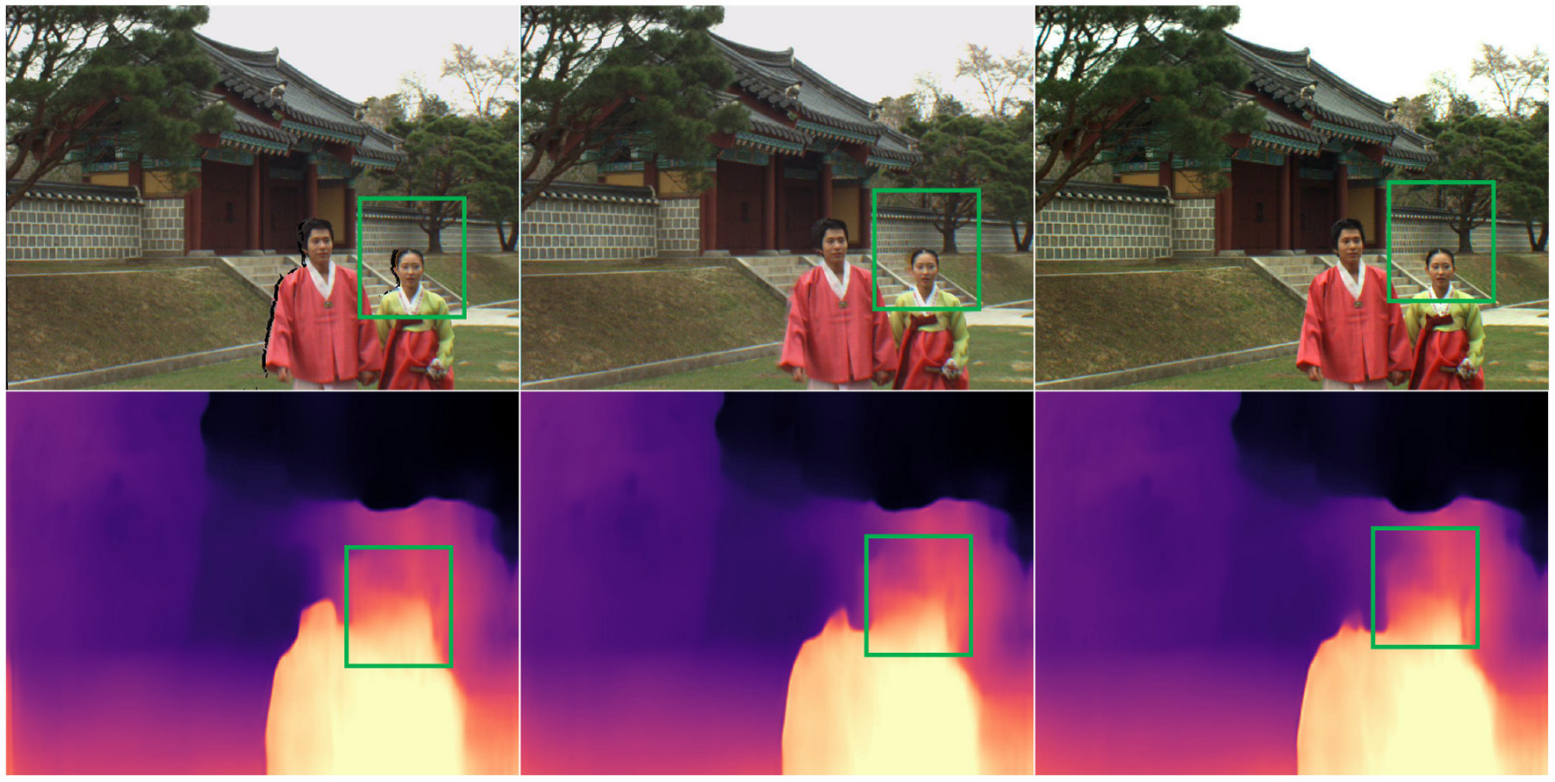
Examples of the depth maps of two synthesized images and their reference image. From top to bottom, the images in each column are a synthesized/reference image and its corresponding depth map. Note that the synthesized image of the first column has only the warping process.

Considering that local geometric distortion and global sharpness damage the structural information of the synthesized view in the texture domain and affect the depth structure of the synthesized view. Thus, we measure the structural similarity between the depth maps of a pair of synthesized and reference views in the depth domain to estimate the depth degradation introduced by the local geometric distortion and blur distortion. The depth map prediction algorithm computes the depth map at the virtual viewpoint. At present, massive deep learning-based depth image estimation algorithms have been proposed (Atapour-Abarghouei and Breckon, 2018; Li et al., 2018a; Zhang et al., 2018; Godard et al., 2019). In our TDI, we employ Clément Godard's depth prediction network for estimating the depth maps of the DIBR-synthesized image and its reference image. Figure 4 shows some examples of the depth maps of two synthesized images and their ground-truth image estimated by Clément Godard's method. From the green box area in Figure 4, it can be easily observed that the local geometric distortion is very destructive to the depth structure of the synthesized image. So the geometric distortion contained in a synthesized image can be effectively estimated by measuring the structural similarity between the depth maps of a pair of synthesized and reference images. In particular, the structural similarity between the depth maps of a synthesized image and its reference image is computed as follows:


(5) $$\begin{array}{l} Q_{3}=\frac{1}{N}\overset{N}{\underset{i=1}{\sum }}\left(SSIM\left(D_{syn}\left(i\right),D_{ref}\left(i\right)\right)\right), \end{array}$$

where *D*_*syn*_ and *D*_*ref*_ represent the depth maps of a synthesized image and its reference image predicted by Clément Godard's algorithm. SSIM is an image quality evaluation index based on the structural similarity between the reference and distorted images (Wang et al., 2004; Jang et al., 2019).

### 2.3. Linear Pooling Scheme

To evaluate the visual quality of DIBR-synthesized views more efficiently, this paper extracts three features from the texture and depth domains to estimate the color deviation distortion, the local geometric distortion, and global sharpness. Since the features *Q*_1_, *Q*_2_, and *Q*_3_ are complementary, we propose a novel linear pooling scheme to fuse the texture and depth information to form the final TDI model. A smaller *Q*_1_ value shows the difference between the colorfulness of the synthesized image and its reference image is smaller. That is, the quality of the synthesized image is higher. The *Q*_2_ and *Q*_3_ are the texture and depth structure similarity between a pair of synthesized and reference images, respectively. The values of *Q*_2_ and *Q*_3_ are higher, indicating that the quality of a pair of synthesized and reference views is more similar. That is, the quality of the synthesized image is better. With this fact, a linear pooling scheme is developed to fuse the obtained features, which is defined as follows:


(6) $$\begin{array}{l} S=-\frac{\alpha }{1+\alpha +\beta }\cdot Q_{1}+\frac{1}{1+\alpha +\beta }\cdot Q_{2}+\frac{\beta }{1+\alpha +\beta }\cdot Q_{3}, \end{array}$$

where the parameters α and β are used to adjust the contribution of *Q*_1_, *Q*_2_, and *Q*_3_. In section 3, we detail the selection of parameters α and β.

## 3. Experimental Results and Discussions

In this part, we construct experiments on the IRCCyN/IVC database to test the performance of the proposed TDI method and other SOTA IQA algorithms.

### 3.1. Experimental Setup

#### 3.1.1. Competing IQA Metrics

In this paper, we collect twenty SOTA IQA algorithms for natural images and DIBR-synthesized images as competing algorithms. The competing IQA metrics designed for natural images include PSNR, SSIM (Wang et al., 2004), IW-SSIM (Wang and Li, 2011), ADD-SSIM (Gu et al., 2016), PSIM (Gu et al., 2017a), NIQE (Mittal et al., 2013), ILNIQE (Zhang et al., 2015), ARISM (Gu et al., 2015), and BIQME (Gu et al., 2018b). The competing IQA methods devised for DIBR-synthesized images consist of MW-PSNR (Dragana et al., 2015b), MP-PSNR (Dragana et al., 2015a), MP-PSNR-reduce (Dragana et al., 2016), NIQSV+ (Tian et al., 2018), APT (Gu et al., 2018a), CLGM (Yue et al., 2019), STD (Wang et al., 2021), LMS (Zhou et al., 2019), IDEA (Li et al., 2021b), GANs-NRM (Ling et al., 2020), and OUT (Jakhetiya et al., 2019).

#### 3.1.2. Testing Dataset

In this paper, we test the performance of the proposed TDI metric and twenty SOTA IQA algorithms on the public IRCCyN/IVC database (Bosc et al., 2011). The IRCCyN/IVC DIBR-synthesized image database contains 12 reference images and its corresponding 84 synthesized images generated via seven DIBR algorithms. In the subjective experiment, the authors adopt the absolute category rating-hidden reference method to mark DIBR-synthesized images. The images in the IRCCyN/IVC image dataset are from three free-view sequences (i.e., “Book Arrival,” “Lovebird,” and “Newspaper”) with a resolution of 1,024 × 768.

#### 3.1.3. Performance Benchmarking

In this paper, three commonly used indicators, including Spearman Rank-order Correlation Coefficient (SRCC), Pearson Linear Correlation Coefficient (PLCC), and Root Mean Square Error (RMSE), are used to evaluate the performance of the proposed TDI metric and other competing IQA algorithms devised for natural images and DIBR-synthesized images. The SRCC index evaluates the monotonic consistency between subjective scores and objective scores predicted by IQA metrics. The PLCC and RMSE indicators evaluate the accuracy of the scores predicted by IQA algorithms. The larger values of SRCC and PLCC, and the smaller value of RMSE, indicate the performance of the corresponding IQA metric is better. The PLCC is defined as follows:


(7) $$\begin{array}{l} PLCC=\frac{\underset{i}{\sum }\left(a_{i}-ā\right)\left(l_{i}-\bar{l}\right)}{\sqrt{\underset{i}{\sum }{\left(a_{i}-ā\right)}^{2}\underset{i}{\sum }{\left(l_{i}-\bar{l}\right)}^{2}}}, \end{array}$$

where *a*_*i*_ and ā are the estimated quality score of the *i*-th synthesized image and the average value of all *a*_*i*_, respectively. *l*_*i*_ and $\bar{l}$ are the subjective quality label of the *i*-th synthesized image and the average value of all *l*_*i*_, respectively. The SRCC is computed as follows:


(8) $$\begin{array}{l} SRCC=1-\frac{6\overset{Q}{\underset{q=1}{\sum }}d_{q}^{2}}{Q\left(Q^{2}-1\right)}, \end{array}$$

where *Q* is the number of pairs of predicted quality scores and subjective quality labels. *d*_*q*_ represents the ranking difference between the predicted quality scores and the subjective quality labels in each group. Before calculating the above indicators, we need to map the quality scores of all IQA methods to the same range through a non-linear logistic function (Min et al., 2020a,b), which is defined as follows:


(9) $$\begin{array}{l} f\left(x\right)=\tau_{1}\left(\frac{1}{2}-\frac{1}{1+e^{\tau_{2}\left(x-\tau_{3}\right)}}\right)+\tau_{4}x+\tau_{5}, \end{array}$$

where τ_1_, τ_2_, τ_3_, τ_4_, and τ_5_ are the fitting parameters. *x* and *f*(*x*) are the quality scores predicted by IQA algorithms and their corresponding non-linear mapping results, respectively.

### 3.2. Performance Comparisons With SOTA IQA Metrics

As shown in Table 1, our TDI metric achieves SRCC value of 0.7905, PLCC value of 0.7992, and RMSE value of 0.4002 on the IRCCyN/IVC dataset, which outperforms most competing IQA metrics designed for natural images and DIBR-synthesized images. In terms of SRCC, the performance of our proposed method is very close to that of the best-performing GANs-NRM. From Table 1, we observe two important conclusions:

1. The performance of the IQA algorithms for natural images on IRCCyN/IVC is far inferior to the IQA methods designed for DIBR-synthesized images. The SRCC, PLCC, and RMSE values of the best BIQME (Gu et al., 2018b) on the IRCCyN/IVC dataset (Bosc et al., 2011) are 0.6770, 0.7271, and 0.4571, respectively, and its SRCC value still does not reach 0.7. Regarding SRCC, PLCC and RMSE, the proposed TDI metrics are 16.77, 9.92, and 12.45% higher than the top BIQME methods, respectively.
2. The APT (Gu et al., 2018a) and OUT (Jakhetiya et al., 2019) metrics, existing best performing IQA algorithms on the IRCCyN/IVC (Bosc et al., 2011) database based on geometric distortion quantization, achieve SRCC value of 0.7157, PLCC value of 0.7678, and RMSE value of 0.4266, respectively. Our proposed TDI metric increases the values of SRCC, PLCC, and RMSE by 10.45, 4.09, and 6.19% on this result. Experiments show that the proposed TDI metric, combining colorfulness, texture structure, and depth structure, can efficiently predict DIBR-synthesized image quality.

**Table 1:** Performance comparison of 21 SOTA IQA measures on the IRCCyN/IVC database (Bosc et al., 2011).

**Metric**	**Type**	**SRCC**	**PLCC**	**RMSE**
PSNR	Natural Images	0.3095	0.3976	0.6109
SSIM (Wang et al., 2004)	Natural Images	0.4368	0.4850	0.5823
IW-SSIM (Wang and Li, 2011)	Natural Images	0.4053	0.5831	0.5409
ADD-SSIM (Gu et al., 2016)	Natural Images	0.4672	0.5512	0.5556
PSIM (Gu et al., 2017a)	Natural Images	0.4576	0.5315	0.5640
NIQE (Mittal et al., 2013)	Natural Images	0.3739	0.4374	0.5987
IL-NIQE (Zhang et al., 2015)	Natural Images	0.5348	0.4998	0.5767
ARISM (Gu et al., 2015)	Natural Images	0.3728	0.3994	0.6104
BIQME (Gu et al., 2018b)	Natural Images	**0.6770**	**0.7271**	**0.4571**
MW-PSNR (Dragana et al., 2015b)	DIBR-synthesized Images	0.5757	0.5622	0.5506
MP-PSNR (Dragana et al., 2015a)	DIBR-synthesized Images	0.6227	0.6174	0.5238
MP-PSNR-reduce (Dragana et al., 2016)	DIBR-synthesized Images	0.6634	0.6772	0.4899
NIQSV+ (Tian et al., 2018)	DIBR-synthesized Images	0.6668	0.7114	0.4679
APT (Gu et al., 2018a)	DIBR-synthesized Images	0.7157	0.7307	0.4546
CLGM (Yue et al., 2019)	DIBR-synthesized Images	0.6528	0.6750	0.4620
STD (Wang et al., 2021)	DIBR-synthesized Images	0.7729	0.7901	0.4082
LMS (Zhou et al., 2019)	DIBR-synthesized Images	0.8050	0.7690	0.3940
IDEA (Li et al., 2021b)	DIBR-synthesized Images	—	0.7796	—
GANs-NRM (Ling et al., 2020)	DIBR-synthesized Images	**0.8070**	**0.8260**	**0.3860**
OUT (Jakhetiya et al., 2019)	DIBR-synthesized Images	0.7036	0.7678	0.4266
TDI (Pro.)	DIBR-synthesized Images	**0.7905**	**0.7992**	**0.4002**

*The best performance in each type is highlighted in bold*.

### 3.3. Ablation Study

**Figure 5:**
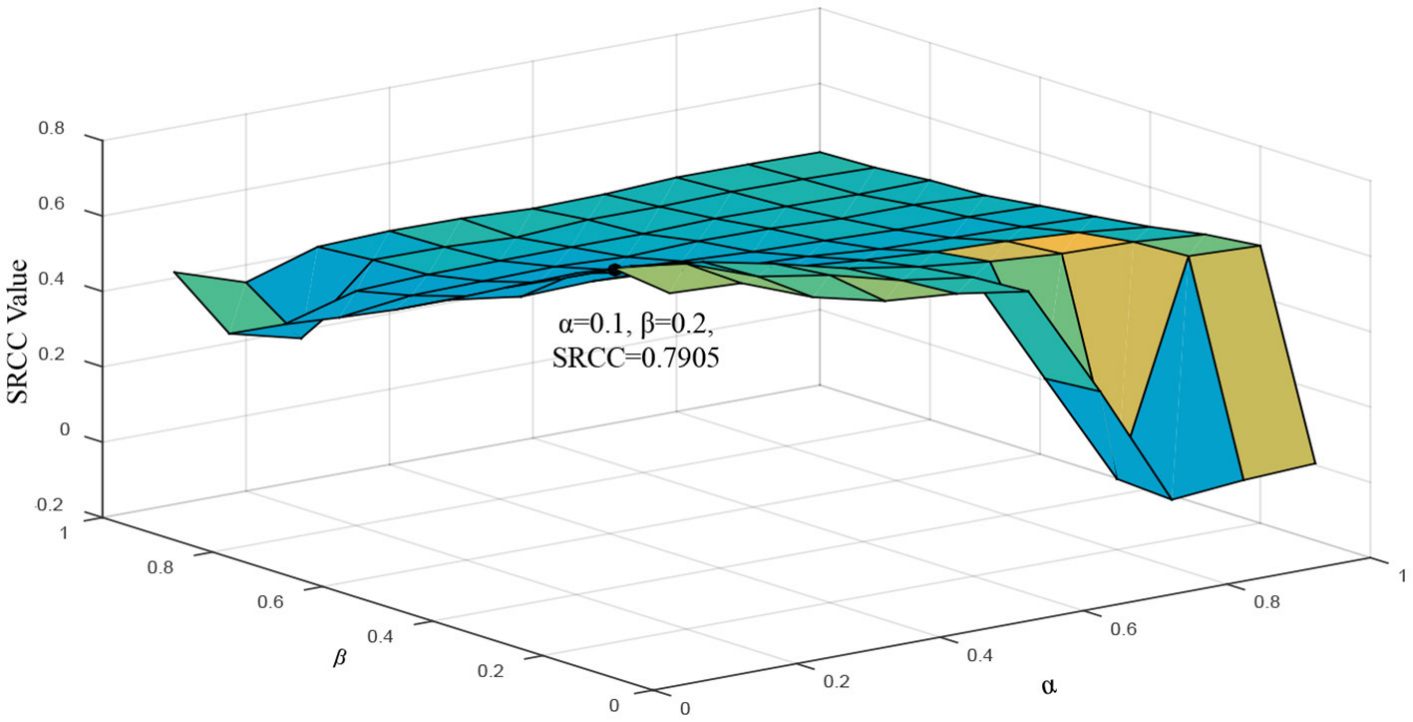
The impact of the parameters α and β on the robustness of the proposed TDI metric.

In this part, we conduct some ablation experiments to verify the contributions of the proposed key components (i.e., *Q*_1_, *Q*_2_, and *Q*_3_). Table 2 shows the test results of the components *Q*_1_, *Q*_2_, *Q*_3_, and the overall module on the public IRCCyN/IVC data set. From the results, we observe the performance of the overall TDI model is far superior to each component, which shows that the proposed sub-modules can complementally evaluate the quality of the synthesized view. That is, the fusion of texture and depth information is of great significance to the view synthesis quality perception. Moreover, we further analyze the influence of the parameters α and β in equation (6) on the robustness of the proposed TDI metric, and the experimental results are shown in Figure 5. Obviously, when the parameters α and β are smaller, the performance of the proposed TDI metric is better, that is, compared to the components *Q*_1_ and *Q*_3_, the component *Q*_2_ is more important, which is also in line with the test results in Table 2. According to the robustness analysis, the parameters α and β are set to 0.1 and 0.2, respectively, to optimize the proposed TDI module.

**Table 2:** Ablation experiments about the proposed components.

**Metric**	**SRCC**	**PLCC**	**RMSE**
Q1	0.4412	0.4971	0.5777
Q2	0.6126	0.6133	0.5259
Q3	0.4470	0.5346	0.5627
TDI (overall model)	0.7905	0.7992	0.4002

### 3.4. Applications in Other Fields

With the rapid development of computer vision, the three-dimensional-related technologies can be implemented in numerous practical applications. The first application is abnormality detection in industry, especially the smoke detection in industrial scenarios which has received an amount of attention from researchers in recent years (Gu et al., 2020b, 2021b; Liu et al., 2021). The process of abnormality detection relies on images, therefore combining three-dimensional technology with this can make the image acquisition equipment obtain a more accurate, intuitive and realistic image information, so as to enable the staff to monitor the abnormal situation in time and then avoid bad things from happening. The second application is atmospheric pollution monitoring and early warning (Gu et al., 2020a, 2021a; Sun et al., 2021). The three-dimensional visualized images contain more detailed information, thus enabling efficient and accurate air pollution monitoring. The third application field is three-dimensional vision and display technologies (Gao et al., 2020; Ye et al., 2020). Compared with the ordinary two-dimensional screen display, three-dimensional technology can make the image is no longer confined to the plane of the screen (Sugita et al., 2019), as if it can come out of the screen, so that the audience has a feeling of immersion. The fourth application is road traffic monitoring (Ke et al., 2019). Three-dimensional technology can monitor the traffic flow information of major intersections in an all-round and intuitive way. All in all, there are several advantages of DIBR technology, so it is necessary to extend this technology to different fields.

## 4. Conclusion

This paper presents a novel DIBR-synthesized image quality assessment algorithm based on texture and depth information fusion, dubbed as TDI. First, in the texture domain, we evaluate the visual quality of the synthesized images by extracting the differences in colorfulness and HH wavelet subband between the synthesized image and its reference image. Then, in the depth domain, we estimate the impact of the local geometric distortion on the quality of the synthesized views by calculating the structural similarity between the depth maps of a pair of synthesized and reference views. Finally, a linear pooling model is developed to fuse the above features to predict DIBR-synthesized image quality. Experiments on the IRCCyN/IVC database show that the proposed TDI algorithm outperforms each sub-module and most competing SOTA image quality assessment methods designed for natural and DIBR-synthesized images.

## Data Availability Statement

The original contributions presented in the study are included in the article/supplementary material, further inquiries can be directed to the corresponding author.

## Ethics Statement

Written informed consent was obtained from the individual(s) for the publication of any potentially identifiable images or data included in this article.

## Author Contributions

QS and YS designed and instruct the experiments. GW wrote the code for the experiments. GW, QS, and LT carried out the experiments and wrote the manuscript. YS and LT collected and analyzed the experiment data. All authors listed have made a substantial, direct and intellectual contribution to the work, and approved it for publication.

## Funding

This research was funded by the National Natural Science Foundation of China, grant no. 61771265.

## Conflict of Interest

The authors declare that the research was conducted in the absence of any commercial or financial relationships that could be construed as a potential conflict of interest.

## Publisher's Note

All claims expressed in this article are solely those of the authors and do not necessarily represent those of their affiliated organizations, or those of the publisher, the editors and the reviewers. Any product that may be evaluated in this article, or claim that may be made by its manufacturer, is not guaranteed or endorsed by the publisher.
